# The Impact of Diet and Physical Activity on Psoriasis: A Narrative Review of the Current Evidence

**DOI:** 10.3390/nu15040840

**Published:** 2023-02-07

**Authors:** Ewa Duchnik, Joanna Kruk, Aleksandra Tuchowska, Mariola Marchlewicz

**Affiliations:** 1 Department of Aesthetic Dermatology, Pomeranian Medical University, Powstańców Wielkopolskich 72, 70-111 Szczecin, Poland; 2 Faculty of Physical Culture and Health, University of Szczecin, Piastów 40b/6, 71-065 Szczecin, Poland; 3 Department of Dermatology and Venereology, Pomeranian Medical University, Siedlecka 2, 72-010 Police, Poland

**Keywords:** psoriasis, quality of life, nutrition, diet, nutrigenetics, physical activity

## Abstract

Psoriasis is an inflammatory disease with strong genetic links and numerous features of autoimmunity that are also influenced by environment and lifestyle, including nutritional factors and physical activity (PA), with regards to the condition of patients. Recent reports in the field of nutrigenomics indicate a significant impact of nutrients in modulating microRNAs. However, few studies have evaluated the effect of nutritional systems and PA on treating psoriasis. This narrative review updates information regarding the current dietary recommendations for individuals with psoriasis and discusses the role of diet and PA in psoriasis prevention and treatment. Application of nutrigenetics in psoriasis therapy is also discussed. The PubMed and Google Scholar databases were searched using the MeSH terms for “nutrigenomics”, “dietetics”, “diet therapy”, “diet”, “physical activity”, and “exercise” in conjunction with the MeSH terms for “psoriasis” and “dermatology”. Evidence has shown that patients with psoriasis should have a personalized anti-inflammatory diet. Psoriasis patients are less physically active; most performed exercises of low-to-moderate intensity and were less likely to undertake regular exercise. Identifying nutrigenomic discoveries and the current lifestyle interventions associated with psoriasis can help physicians and physical therapists develop educational programs to manage and protect against the disease.

## 1. Introduction

The skin is an organ that protects against environmental stressors and plays a fundamental role in cellular signaling and maintaining homeostasis [1]. The skin is continuously exposed to free radicals, reactive oxygen species (ROSs), and nitrogen species (RNSs), such as superoxide anion radicals (O_2_^●−^), hydrogen peroxide (H_2_O_2_), singlet oxygen oxygen(^1^O_2_), hydroxyl radicals, nitrogen monoxide (NO^●^), hypochlorous acid (HOCl), and peroxynitrite (ONOO^−^) attacks. When these species are excessively generated and the potency of antioxidant defense systems is insufficient, the cellular redox state can be shifted towards oxidizing conditions, followed by a disruption of redox homeostasis and inflammation (oxidative stress, OS, conception) [2]. Psoriasis is a common chronic disease of unclear etiology. It has strong chronic inflammatory genetic links and numerous features of autoimmunity, which can occur in patients of any age [3]. Psoriasis is influenced by lifestyle factors, such as nutritional and physical activity (PA). This disease is estimated to affect approximately 2% of the world’s population, negatively impacting the quality of life [3,4]. The clinical picture of psoriasis is heterogeneous due to the presence of many clinical varieties of this disease [5,6]. The most common type of psoriasis is psoriasis vulgaris (approximately 90% of cases) [6]. This type of the disease is characterized by sharply delineated erythematous itchy skin lesions covered with silvery scales [5,6,7]. Psoriatic lesions are characterized by hyperproliferation, incomplete differentiation of epidermal keratinocytes, and decreased keratinocyte apoptosis associated with the dermis and epidermis inflammatory infiltration. However, psoriasis is not a disease confined to the skin. In modern medicine, it is considered a systemic disease. The disease is closely related to metabolic disorders, such as insulin resistance, atherogenic dyslipidemia, hypertension, increased body mass index (BMI), obesity, and cardiovascular diseases [6]. It also can affect the joints (psoriatic arthritis) and increase the risk of kidney disease, digestive tract disorders, and excessive mortality [7]. Recent reports also suggest a correlation between the occurrence of psoriasis and inflammatory bowel disease (IBD), celiac disease, non-alcoholic fatty liver disease (NAFLD), uveitis, osteoporosis, and depressive disorders [6]. Hence, psoriasis is less and less often called a dermatological disease. The literature has documented common mechanisms between physical inactivity and inflammatory disease, including psoriasis, such as increased adiposity, inflammation, OS, adhesion molecules, and lipid peroxidation. In addition, epigenetic processes can be modulated by PA [8]. Therefore, a vital element of holistic care for patients with psoriasis is education in the use of an appropriate diet and regular moderate physical activity (PA) to protect against the development of the above diseases [4,5]. Getting to know the sequence of the human genome had a decisive influence on the direction of the development of biological sciences. In recent years, there has been significant development not only of genomics but also of disciplines combining the science of food and nutrition with molecular biology: nutrigenomics (the science that studies the effects of nutrients on genome function in terms of gene transcription, protein levels, and epigenetic mechanisms) and nutrigenetics (the science that focuses on genetically determined differences in the metabolic response to individual components of the diet) [9,10]. Better understanding of these relationships supports the individualization of nutrition and the possibility of preventing and treating many chronic diseases. This review aimed to present the current dietary recommendations and new options for supporting the treatment of psoriatic patients through proper nutrition and engaging in PA. The review attempts to present types of diet in the treatment of psoriasis and their effectiveness. We have also attempted to provide insight into the development of nutrigenetics and nutrimiromics in this area. Furthermore, the current evidence for the preventive action of physical exercise (PE) against psoriasis and its role in disease treatment will be discussed. Due to the large body of the subject literature, we present a representative cross-section of the findings in this field.

## 2. Materials and Methods

The PubMed and Google Scholar databases were searched for relevant publications. The search was performed using the Medical Subject Headings (MeSH) terms for “nutrigenomics”, “dietetics”, “diet therapy”, and “diet” in conjunction with the MeSH terms for “psoriasis” and “dermatology”. For elucidation of the psoriasis–PA association, the following keywords were used: the MeSH terms for “psoriasis” OR “dermatology” AND “physical activity” OR “physical exercise” OR “sports”. Initial screening of articles evaluated the title and abstract of the paper. A secondary screening evaluated the studies for relevancy to remove duplicates. The review was limited to studies published in English between January 2012 and November 2022. Studies were included in this narrative review if they met the following criteria: they (a) were original human studies, reviews, or reviews and meta-analyses, (b) examined the association between PA and risk of psoriasis, (c) provided relative risk or adds risk (RR/OR) and 95% confidence intervals (CIs) or quantification of the impact, and were adjusted for confounders or matched. The article mainly focuses on recently published reviews and observational studies. However, a few earlier published original studies also have been presented in Table 1 as representative findings.

## 3. Results and Discussion

### 3.1. Psoriasis and Its Connection with Genes

So far, the strongest association with the risk of psoriasis development has been shown for the human leukocyte antigen (HLA)-T allele HLA-C × 06 genes [11]. Most of the single nucleotide polymorphisms of other types of psoriasis risk are found near the genes that code for molecules involved in adaptive and innate immunity and skin barrier function. Researchers indicated that epigenetic changes such as promoter methylation, histone modification, long non-coding microRNA (miRNA, miR), and its overexpression contribute to the development of psoriasis. It is due to aberrant regulation of keratinocyte proliferation and differentiation, neoangiogenesis, and chronic inflammation [5]. MicroRNAs are small non-coding RNA molecules involved in RNA silencing and post-transcriptional regulation of gene expression [12]. They also appear to mediate immune dysfunction in psoriasis. Although microRNA research is a new field in dermatology, evidence rapidly accumulates that it contributes significantly to the pathogenesis of chronic inflammation, including psoriasis and other dermatological diseases [13,14]. In addition, circulating miRNAs found in patients’ blood samples have been identified as promising biomarkers for diagnosis, prognosis, or treatment response [12]. 

Different molecules associated with various miRNA modifications in psoriasis have been identified. The main miRNAs linked to this disease are: miR-21, miR-31, miR-136, miR-143, miR-146, miR-155, miR-203, miR-221/2, miR-223, Has-miR-99a, miR-125b, miR-138, and miR-424 [12,15]. Circulating miRNAs detected in the blood may constitute specific markers in the diagnosis, prognosis, and response to treatment of the disease. It has been shown that the inhibition of, for example, miR-21, miR-31, miR-146a, and miR-155 shows significant therapeutic effects in the treatment of psoriasis by reducing inflammation [14]. Some compounds that can be delivered to the body with food have such abilities. The discoveries in this field were so important that a separate research area was established—nutrimiromics, which studies the influence of diet on the modification of gene expression due to epigenetic processes related to miRNA [14]. 

### 3.2. Nutrients and Dietary Components in Psoriasis

In psoriasis etiology, a unique role is played by the induction of inflammation with the activation of T-helper (Th-1) and Th-17 lymphocytes, leading to increased production of inflammatory cytokines, such as interleukins: IL-1, IL-6, IL-23, IL-22, IL-17, and IL-33, tumor necrosis factor-alpha (TNF-α), and interferon-gamma (IFN-γ). However, nutrition may modulate inflammation due to the presence of antioxidant and anti-inflammatory components [16]. Lipids play a special role in modulating inflammation. Saturated fats (SFAs) found, among others, in butter and red meat increase the concentration of interleukins and contribute to the development of inflammation [17]. Similarly, the pro-inflammatory effect may also be exerted by an excess of unsaturated fatty acids from the *n*-6 family, present, for example, in margarine and refined vegetable oils [18]. For good health, foods high in monosaturated and polyunsaturated fatty acids (PUFAs), such as plant-based oils, nuts, and fish, are recommended [18,19]. There are two main groups of these essential biomolecules: omega-3 (α-linoleic acid, eicosapentaenoic acid (EPA), and docosahexaenoic acid, *n*-3) and omega-6 (linoleic acid, arachidonic acid (AA), *n*-6). The human body does not produce both PUFAs, which must be obtained from the diet [20]. Vegetable oils, such as corn oil, sunflower oil, soya bean oil, cotton seeds, grains, and corn, are rich in *n*-6 PUFAs, whereas flaxseed oil, canola/rapeseed oil, perilla oil, and chia oil are rich in *n*-3 PUFAs. Seafood and fish, especially marine fish, are recognized as the essential sources of *n*-3 PUFAs [19,20]. The *n*-3 and *n*-6 PUFAs are essential components of cell membranes and precursors of signaling molecules involved in regulating metabolic pathways and blood pressure, as well as influencing gene expression. These compounds are essential for brain function and cell growth, playing important roles in inflammation and modulation of immunity. Their levels may be related to the pathogenesis of many diseases [21,22]. The *n*-3 and *n*-6 PUFAs compete for the same metabolic enzymes, and their biotransformation to prostaglandins, thromboxane, and leukotrienes may lead to an increased inflammatory state and disturbance in energy homeostasis [19]. However, *n*-3 and *n*-6 PUFAs exert opposing effects on metabolism [21,22]. Therefore, the levels of PUFAs ingested should be balanced because an imbalance may change cell membrane properties and function, promoting an inflammatory environment [22]. The *n*-6/*n*-3 and AA/EPA PUFA ratios are useful biomarkers which may provide crucial information on nutritional needs, health, and disease risk [19,21,22]. Intake of 1.1–1.6 g/day of *n*-3 and *n*-6/*n*-3 PUFA ratios between 4/1 and 7.5/1 in the diet have been recommended as optimal, considering the prevention of chronic diseases and their management [19]. However, a typical Western diet increases the ratio of *n*-6/*n*-3 PUFAs to between 10/1 and 20/1. A very high *n*-6 to *n*-3 ratio has been reported in several studies as a factor in decreasing insulin sensitivity and promoting inflammatory and immune diseases [19]. However, scientific evidence in this respect has shown conflicting findings, likely due to the research methodology. Thus, PUFAs are not fully utilized in clinical applications [22,23,24].

Evidence has maintained that therapeutic doses of PUFAs may depend on several factors, such as endogenous metabolism of linoleic acids, diet, the individual’s redox state, severity and type of disease, and PA. Despite the contracting results, the current evidence has suggested that AA/EPA ratio and *n*-3 are promising biomarkers for PUFA metabolism [22]. Simple sugars may also exacerbate inflammation in psoriasis [25,26]. It is probably related to excessive consumption of carbohydrates and the simultaneous development of anthropometric changes, including increased body weight and waist circumference, predisposing diseases such as obesity or cardiovascular diseases. A link was found between lowering the glycemic index (GI) and reducing inflammation [27]. Some studies showed a beneficial effect of dietary intervention in psoriasis, resulting in lowering glycaemia and postprandial insulinemia, which, in turn, prevents the development of other inflammatory diseases of civilization. Researchers also indicated the enhancement of pharmacological therapy using TNF-alpha inhibitors, for example, polyphenols in combination with a low-carbohydrate diet [28]. However, a systematic review and meta-analysis by Milajerdi et al. [29] did not confirm a significant effect of the glycemic index (GI) or glycemic load on serum levels of inflammatory cytokines.

A vital element of the nutrition of people suffering from psoriasis is anti-inflammatory and antioxidant compounds, e.g., dietary fibers, omega-3 acids, some polyphenols, vitamins A, E, and C, and oligo-elements (copper, manganese, zinc, and selenium) [18,30,31,32]. The most frequently reported dietary components that improve the condition of patients include fish oil (omega-3 polyunsaturated fatty acids), fruits and vegetables, and vitamin D and probiotics supplementation [26,33,34]. The effect of vitamin D on the skin is very complex [31,32]. The active form of vitamin D, calcitriol (also known as 1,25-dihydroxycholecalciferol), and its receptor regulate the differentiation and proliferation of keratinocytes, the balance of the dermal immune system, and apoptosis. In addition, 1,25(OH)_2_ D and analogues lower the level of psoriasis (S100A7), which is increased in the psoriatic skin. Evidence exists that 1,25(OH)_2_ D has an antiproliferative effect on keratinocytes [31]. A decrease or a deficiency of 1,25(OH)_2_ D disrupts epidermal differentiation, reduces the level of involucrin and loricrin, and causes a loss of keratohyalin granularity, which leads to hyperproliferation of the basal layer. In most patients, the severity and duration of psoriasis are positively correlated with serum vitamin D deficiency [18,32]. 

Researchers also pay attention to the role of gut microbiota in inflammatory skin diseases [33,34,35,36]. Probiotics and prebiotics are indispensable elements of a well-composed diet for patients with psoriasis [36]. The gut microflora and skin microflora play an essential role in various aspects of the disease, from pathogenesis to response to treatment. Research confirms a strong and bidirectional correlation between the gut and skin, linking digestive health with skin homeostasis and allostasis. For example, 7–11% of patients with inflammatory bowel disease (IBD) are also diagnosed with psoriasis, indicating a strong association with gastroenteritis [37]. It has been proven that some genetic and environmental factors and immune pathways may be involved in the etiopathogenesis of both diseases. Among others, Th17 cells and their cytokines play an essential role in developing psoriasis and the pathophysiology of IBD [38]. Moreover, the same pattern of dysbiosis is observed in both IBD and psoriasis [39]. It is characterized by the depletion of symbiont bacteria and colonization by some pathobionts. Colonization of the skin or the intestines (or both) by Staphylococcus aureus, Malassezia furfur, and Candida albicans exacerbates psoriasis. Moreover, the decreased abundance of two beneficial bacterial species, Parabacteroides and Coprobacillus, is also noticeable. It can lead to deleterious consequences, including poor regulation of the intestinal immune response, which, in turn, can affect distant organ systems. That is why scientists are increasingly recommending probiotic supplementation to treat psoriasis. Reports from Murine and human models indicated suppression of psoriasis-related TNF-alpha and IL-6 among other pro-inflammatory cytokines in the cytokine axis IL-23/IL-17 after supplementation with probiotics, among others [33,35,39]. 

Researchers also point to the participation of other food ingredients with the potential to inhibit the development of psoriasis. An example here is cyclitols (sugar alcohols) [40]. Their immunomodulatory effects may suggest that some of them, such as D-pinitol, D-chiro-inositol, and myo-inositol, may be used to treat psoriasis. These compounds can normalize the balance between Th1, Th17, and Th2 lymphocytes, inhibit the production of pro-inflammatory cytokines (e.g., TNF-α), stimulate keratinocyte apoptosis, and inhibit oxidative stress (OS) and angiogenesis. Moreover, the popular substitutes for white sugar, such as xylitol or steviol, positively affect the oral cavity and intestinal microbiota, which may also indirectly improve the skin’s condition in many dermatoses [34,41,42]. 

There are also indications that genistein (a flavonoid, an antioxidant found, among others, in soybeans) positively reduces inflammation in psoriasis [43]. A study in mice with psoriasis showed that the administration of genistein reduced skin lesions in the histological image. On the other hand, genistein administered to patients changed gene expression in psoriatic skin lesions, inhibiting the disease’s progression [43,44]. 

Scientists also pay particular attention to an essential role of the trace element selenium in psoriasis diet therapy. Selenium occurs in the active center of glutathione peroxidase and plays a vital role in a cellular antioxidant defense system. The element inhibits ROS and deactivates the nuclear factor kappa-B (Nf-κB) redox signaling pathway, correlated with IL-6 and TNF-α generation and inflammation [45]. People with psoriasis often have a low level of selenium in the serum. However, there are no clear reasons to recommend additional selenium supplementation due to limited evidence confirming its therapeutic efficacy [46]. The gut microbiome has recently been a subject of scientists’ interest. Evidence shows that some psoriasis patients also note the presence of so-called “triggers” of lesions, which worsen their psoriasis [26]. The most common declared triggers are: white sugar, alcohol, nightshade vegetables (tomatoes, potatoes, eggplants, peppers), gluten, dairy products, meat, and processed foods. The negative impact of these dietary components is explained by the fact that they can cause changes in the composition of the gut microbiome, irritate the gut epithelium, and cause dysregulation of the immune system [26]. Simple sugars in the diet lead to dysbiosis of the intestinal microbiome, favoring harmful bacterial taxa and increasing the concentration of inflammatory cytokines. However, high-fiber complex carbohydrates from fruits and vegetables have the opposite effect on the gut microbiome. They can reduce inflammation. In turn, the harmful effect of alcohol in psoriasis is caused by enhancing mitogen-dependent lymphocyte proliferation and upregulation of pro-inflammatory cytokines [47]. Fruits and vegetables provide a wealth of antioxidants, such as carotenoids, flavonoids, vitamins, and minerals, that have been inversely correlated with TNF-alpha, C-reactive protein (CRP), and IL-6 generation. However, in this food group, consumption should be aware of the above-reported exceptions because they can disrupt the intestinal lining, producing alkaloids. In a mouse model, they were found to adversely affect mammals’ intestines and aggravate inflammatory bowel disease (a frequent comorbidity in psoriasis patients) [26,47]. 

### 3.3. Types of Diet and Nutritional Systems in the Treatment of Psoriasis

In terms of supporting the treatment of psoriasis with proper nutrition, studies have shown a positive effect of low-energy and vegetarian diets, weight loss programs with a modified diet, a gluten-free diet, a Mediterranean diet, or a very low-calorie diet without carbohydrates [48]. These diets usually have a high proportion of vegetables and fruits; thus, the patient is provided with less energy with a simultaneously ample supply of vitamins, minerals, and other health-promoting compounds [48]. The Mediterranean diet is rich in healthy foods, such as fresh vegetables and fruits, legumes, seeds, olive oil, and fish, with a reduced supply of simple carbohydrates and animal fats. Researchers point to the positive effect of the Mediterranean diet on inflammatory diseases, including psoriasis [49]. One of the most recognized hypotheses is that the high content of antioxidants including polyphenols, largely present in Mediterranean foods (plant foods, fruits, and red wine), has anti-inflammatory and antioxidant properties. Consumption of monounsaturated fatty acids has health benefits in reducing the risk of coronary heart disease, preventing several types of cancer, modifying the immune and inflammatory response, and reducing the risk of osteoporosis [49]. Beta-carotenoids, polyphenols, folic acid, and fibers specific to this diet have been suggested to play a crucial role in preventing oxidative stress [30,50,51]. Various studies have shown that single foods typical of the Mediterranean diet, such as fruit, vegetables, whole grains, and sea fish, are associated with lowering the most common inflammation marker CRP [48,52]. In contrast, after consuming high-energy, nutrient-poor, and processed foods, meal-induced inflammation was confirmed by increased CRP levels immediately [49,53]. 

Another option for people with psoriasis is the ketogenic diet. It is a dietary regimen characterized by a reduction in carbohydrate intake and a relative increase in protein and fat presence on the menu [54]. On a biochemical level, the ketogenic diet causes a shift to ketone metabolism, forming ketones from fat as the main energy source, followed by a decrease in glucose levels and increasing blood ketones. Studies have shown that using a low-calorie ketogenic diet in patients with psoriasis decreased the concentration of pro-inflammatory cytokines (including IL-2 and IL-1β). It also decreased the PASI index (psoriasis severity assessment scale, Psoriasis Area and Severity Index) [54,55]. 

Some literature data suggest a gluten-free diet’s therapeutic role in treating psoriasis [48]. However, current reports do not support the hypothesis regarding the beneficial role of this type of diet in psoriasis therapy. Some patients with psoriasis often have elevated anti-gliadin antibodies IgA and IgG [56]. It may suggest coexisting celiac disease [57]. Therefore, a gluten-free diet should only be used by people with confirmed gluten intolerance [48,57,58]. 

A vegetarian diet, also offered to patients with psoriasis, is characterized by the supply of many legume seeds, various types of cereals and groats, vegetables, fruits, nuts, and mushrooms [48]. Depending on the variation of the vegetarian diet, the consumption of dairy products and eggs is also allowed. This diet is low in saturated fatty acids and cholesterol due to eliminating meat. It is essential in preventing cardiovascular diseases, regulating uric acid levels, and lowering CRP and triacylglycerols in the blood serum. Additionally, patients who follow a vegetarian diet are less likely to experience excess body weight. The positive effect of a meatless diet is explained by a reduction in polyunsaturated fatty acid, arachidonic acid, and its oxidative metabolism derivatives (leukotriene LTB4). These compounds show a pro-inflammatory effect and are responsible for increasing the production of IL-1β and tissue sensitivity to this cytokine [58]. Moreover, patients following a vegetarian diet have higher concentrations of anti-inflammatory adipokines concerning pro-inflammatory adipokines, weaker expression of pro-inflammatory genes in the intestinal microbiota, and a lower level of IgE expression [48,59]. 

### 3.4. Application of Nutrigenetics in the Psoriasis Therapy

The impact of oral nutraceuticals on skin health benefits has gained growing interest in the past two decades [60]. Dermatonutrigenetics, a branch of nutrigenetics, is in the early stages of development [61]. Dermatonutrigenetics studies genes and nutrition and their relationship to identify their combined effects on skin health. Selected genetic mutations are also tested to determine which nutraceuticals will benefit skin functioning. The most frequently studied mutations involve the enzymes participating in collagen decomposition, elimination of ROS generated in excess, for example, by UV-radiation, degradation of environmental pollutants, and decreasing formation of pro-inflammatory molecules [61,62]. 

The possible effect of nutrients on gene expression and clinical progression or remission of psoriasis has yet to be fully understood. It may be a significant challenge in an approach to adjuvant therapy in psoriasis. Evidence has shown that many dietary factors can benefit the affected skin, while others can exacerbate inflammation and immune responses, thus leading to psoriasis comorbidities. It is also maintained that when specific genetic variants are present, the complex phenotype of the disease requires dietary and lifestyle triggers. Moreover, a putative relationship between the skin, immune system, and nutrients has been revealed. For this reason, the nutrigenomic approach has been used in studying psoriasis’s etiology and treating people suffering from this disease [61].

Psoriasis is treated with compounds that act on pro-inflammatory molecules, such as TNF-α inhibitors. It is observed that every third patient does not respond to treatment [63]. One of the factors responsible for the ineffectiveness may be the previously described nutritional stimuli. Unfortunately, research in nutrigenomics in psoriasis that could elucidate the putative mechanisms involved in the role of nutrients correlating with inflammation is limited. Identifying new biomarkers or patterns that would link the nutritional aspect of psoriasis is one of the significant challenges of the omics sciences [5,63]. 

One of the crucial discoveries of nutrigenomics dealing with the effect of nutrients on genes is the modulation of miRNAs [12]. Researchers have proposed mechanisms that include epigenetic DNA reprogramming and evaluation of stimulated gene expression and mRNA transcription, DNA silencing by methyl-CpG-binding proteins [64], histone modification, and expression of small non-coding RNAs (miRNAs) [5,12,65,66]. MicroRNAs regulate inflammation by modulating inflammatory pathways. Evidence has shown that miR-155 (a non-coding micro-RNA) is involved in cellular signaling, immune regulation, inflammation, and metabolism and is highly correlated with nutrition [67].

Natural antioxidant and anti-inflammatory agents, e.g., polyphenols (e.g., curcumin, quercetin, and resveratrol), coenzyme Q10, vitamin D, and selenium have been documented to modulate miRNA expression in a variety of chronic diseases, including psoriasis [5,65,66].

Dietary modulation of miRNA-21 and miR-155 expression by active dietary compounds was analyzed in vivo and in vitro. For example, flavonoids such as resveratrol and quercetin were reported to be the potent epigenetic modulators of miR-155 expression, followed by inhiation of NF-kB activation and anti-inflammatory action [14,68,69]. Moreover, supplementation with quercetin showed a decrease in the expression of TNF-α, IL-6, and IL-17 and a decrease in the expression of the NF-κB through changes in the expression of miR-146 [5,14,67,69].

Next, curcumin reduced the expression of miR-21 and miR-155 [70], followed by the suppression of the key proliferative kinases AKT kinase, JNK kinase, TOR signaling, and transcription factor AP-1. Inflammation was attenuated by the reduced NF-kB activation by TNF-α and IL-6 cytokines [5,70]. Reduction in the miR-155 and miR-125b levels was also observed after treatment of vitamin D within in vitro and in vivo experiments [71,72]. MiR-125b has been reported to decrease keratinocytes and the peripheral blood of people with psoriasis. The anti-inflammatory effect was achieved through decreased NF-κB signaling and reduction in its subunit p65 [70,73].

### 3.5. Benefits of Physical Activity with a Focus on Inflammation and Oxidative Stress

Physical inactivity, defined as “An insufficient physical activity level to meet present physical activity recommendations” [74], is one of the crucial factors responsible for obesity and abdominal fatness [75]. Fat cells (adipocytes) are a source of pro-inflammatory adipokines (leptin and resistin). They can induce T helper 17 cells secreting pro-inflammatory cytokines (TNF-α and IL-6) and CRP [76]. Obesity, fatness, and physical inactivity are likely critical risk factors for psoriasis development [75]. Evidence has shown that regular physical exercise (PE) of moderate intensity (50–<60% VO_2_max) (e.g., walking, dancing, yoga, badminton, downhill skiing, gardening) is an important factor regulating ROS/RNS levels in cells, protecting them against low levels. The species are essential for the natural metabolic redox-sensitive processes [77]. They are crucial secondary messengers in intracellular signaling, moderate gene and enzyme expression, and participate in cells’ immune response to external stressors, among others [78]. However, ROSs/RNSs are effective oxidants and unbalancing their production can damage DNA, causing disturbance in the signal transduction and gene expression, possibly followed by inflammation and OS, increasing the risk of several diseases [78]. The beneficial effects of regular moderate-intensity PA are achieved through cell adaptation to OS via the enhanced expression of genes involved in antioxidant enzyme generation. Other essential protective mechanisms include the increased level of sex hormone-binding globulin; reduction in IGF-1; decreased production of toxic quinone free radicals (e.g., products of oestrogen metabolism); and reduction in body weight and adipose tissue followed by decreases in insulin, glucose, and leptin levels, limiting contribution to systemic inflammation. In addition, increasing anti-inflammatory adiponectin (a protein hormone involved in fatty acid breakdown and regulation of glucose) levels and up-regulation of the NF-κB signaling pathway were reported [79,80,81,82].

Moreover, regular moderate-to-vigorous PE improves immune system functioning and positively affects the level of monocytes, neutrophils, lymphocytes, and eosinophils. The research literature has shown that PE can reduce the risk of developing at least 35 human health disorders [83]. Exercise has also been shown to positively affect human mental health, reducing psychological stress, anxiety, and depression [84]. On the other side, it is recognized that acute exercise, not preceded by training, can increase the ROS/RNS generation and disturb redox homeostasis, elevating inflammation in the cells and organs and contributing to OS, exerting an adverse effect on psoriasis [10,85].

Due to the physical and mental health, economic, and social benefits of moderate-to-vigorous PA, there is a worldwide increase in promoting PA. According to the World Health Organization (WHO) 2020 guidelines [74], adults (18–64 years) should take up at least 150–300 min/week of regular aerobic PA of moderate intensity (3.0–<6.0 MET), at least 75–150 min/week of vigorous intensity (6.0–≤9 MET)(estimated on a rating scale of 0–10 MET), or an equivalent of combined PA of moderate and vigorous intensities(an activity with a one metabolic equivalent task, MET, is the energy spent sitting at rest). Despite the well-recognized benefits of PA, only two-thirds of adults achieve the WHO recommendations globally [74].

### 3.6. The Role of Physical Exercise in Psoriasis Prevention and Treatment

As regular moderate-to-vigorous PE has essential anti-inflammatory and antioxidative abilities, a significant question is what dose of PE and intensity is needed to reduce the risk of psoriasis. Evidence has long demonstrated that patients with psoriasis take up less intensive PA than those without psoriasis [4,75] and have a higher risk of developing cardiovascular disease, obesity, and metabolic alteration, among others [86]. In recent years, two reviews and meta-analyses have quantified the association of psoriasis with PA [4,75]. A review and meta-analysis by Zheng et al. [4] based on 13 observational studies performed from January 1970 to February 2017 demonstrated that patients with severe psoriasis performed vigorous PE 32% less often than controls. They also were engaged in exercise of moderate intensity 60% more often and undertook regular PE 12% less often, compared to controls. These authors concluded that PA of higher intensity may decrease psoriasis prevalence and that the patients more affected by psoriasis and more aware of their disease undertook lower-intensity exercise. A current review by Yeroushaimi et al. [75], using a data set of 28 clinical trials and observational cross-sectional studies, summarized the literature findings on the differences in the prevalence and levels of PE and participation in sports between psoriasis patients and individuals without psoriasis. Evidence showed that psoriasis patients not only had significantly reduced levels and doses of PA, but also had reduced sports activities and were less likely to engage in an exercise of vigorous intensity (a physical effort needed to lose the body weight), or even to participate in light-to-moderate intensity exercise. The authors found a statistically significant negative correlation between the overall quality of the patient’s life and a moderate or vigorous PE dose. The psoriasis patients declared a side effect linked with exercise soreness, increased purities, and difficulties in disease management. Moreover, skin sensitivity and reluctance to exercise were also declared barriers. They reported that over 50% of patients with psoriasis did not achieve the WHO recommendation of PA. 

Table 1 shows the representative original human studies published between 2012 and 2022 for the preventive role of PA in psoriasis development and compares patients with psoriasis to controls regarding physical activity. 

**Table 1:** The representative observational studies for the association between psoriasis and physical activity.

Study/Year/Country	Study Design/Number Participants	Time Period	Category of Physical Activity and Detection	Main Result: RR/HR/OR, 95%CI	Author’s Conclusion
Frankel et al. 2012, USA [87]	Cohort 86,655 female nurses	1991–2005	Self-reported average time/week of recreational activity: walking, jogging, running, bicycles, swimming, performing calisthenics, aerobic, playing tennis, and others, performed during the preceding year	Decreased adjusted for age RR of psoriasis in the most active quintile vs. least active quintile: RR = 0.72 (95%CI: 0.59–0.89). Adjusting for age, BMI, smoking, and alcohol intake in the analysis did not substantially change the summary risk estimates: RR = 0.73 (0.54–0.81)	Vigorous PA prevents against incident of psoriasis
Balato et al. 2015, Italy [88]	Cross-sectional 400 cases, 498 controls	September 2012–June 2013	Questionnaire identifying sport history: type of practiced sports, frequency, number of years regarding practice of sports	Mean duration of sport activity (years ± SD): psoriasis group 4.0 (10.3), control group 4.2 (9.3) Duration (hours/week): <3 h: cases 4.5% controls 4.2% 3–7 h: cases 3.5% controls 5.8% >7 h: cases 3.0%controls 4.4%	Regular vigorous PA may lower the risk of psoriasis
Torres et al. 2014, Portugal [89]	Case-control 90 cases, 160 controls	NP	TA assessed using the International Physical Activity Questionnaire Short Form	Patients with severe psoriasis were 3.42 times less physically active compared to patients without psoriasis: OR = 3.42 (1.47–7.9). Low-intensity PA: cases 18.9%, controls 6.3% Moderate-intensity PA: cases 51.1%, controls 37.6% High-intensity PA: cases 48.9%, controls 62.4%	Patients with psoriasis undertake lower-intensity PA
Do et al. 2015, USA [90]	Cohort 6549 158 cases, 6011 controls	2003–2006	Responses to survey questions on moderate-to-vigorous leisure-time PA (duration, intensity, frequency in the past 30 days). Standardized measure of intensity in MET-min/week	Participation in leisure moderate-to-vigorous PA: cases 65.5% controls 69.7%; the difference was statistically insignificant. MET-min/week engaging in moderate-to-vigorous PA was lower by 30% for patients having fewer or more cutaneous skin lesions than individuals who were never diagnosed with psoriasis	There is a need to develop an effective measure for severity of psoriasis to increase engaging in PA among psoriasis patients
Goto et al. 2020, Japan [91]	Cohort 487,835 participants, 2793 cases	2012–2018	Self-reported PA (walking and exercise)	Exercise to sweat lightly for less than 1 h/week is associated with the risk of psoriasis: HR = 1.13 (1.05–1.22)	Dietary intervention and PA may reduce psoriasis outcomes and the risk related to systemic inflammation
Enos et al. USA 2022 [92]	Case-control 20 cases, 23 controls	NP	The self-efficacy questionnaire for exercise scale. Accelerometer—assessed PA and two 20 min bouts on a treadmill-measure	No statistically significant difference in duration of exercising between psoriasis patients and healthy controls (mean exercise time 26 (4) min vs. 27 (4)). Patients with psoriasis selected treadmill speeds that were 13–18% slower than controls. At the same time, they experienced more pruritus when exercising	Patients with extensive psoriasis and poorer self-efficacy for exercise take up less exercise and take fewer footsteps
Nowowiejska et al. 2022 [93]	Case-control	NP	International Physical Activity Questionnaire PA: walking, moderate or vigorous, performed during the past 7 days (in MET-min/week)	Statistically significant difference in intensity of PA between cases and controls Median activity: cases 693 MET-min/week controls 2016 MET-min/week Levels of PA cases: 48.2% low, 32.1% moderate, 19.6% high controls: 19.4% low, 47.2% moderate, 33.3% high PA was not correlated with psoriasis area and severity	Individuals with psoriasis undertake lower-intensity PA

Abbreviations: RR, relative risk; HR, hazard ratio; OR, odds ratio; CI, confidence interval; PA, physical activity; MET, metabolic equivalent of task; BMI, body mass index; SD, standard; NP, not provided deviation.

A prospective follow-up 14-year study by Frankel et al. [81] (116,430 female nurses, 1026 females with psoriasis) found a protective effect of PA against the risk of psoriasis development. Psoriasis cases of the highest quintile of PA had a 28% reduced multivariable-adjusted relative risk (RR) of the disease (RR = 0.72, 95% CI: 0.59–0.89) compared with those of the least active quintile. Moreover, vigorous-intensity PA (≥6 MET) was also linked with statistically significant risk reduction (RR = 0.66, 95% CI: 0.54–0.81). The authors concluded that vigorous-intensity PA also could reduce psoriasis risk incidence. A large cohort study by Goto et al. [91] confirmed that dietary intervention and regular PA might reduce severe psoriasis outcomes and the risk of systemic inflammation. The authors observed an increased psoriasis risk of 13% among individuals with low activity.

In an experimental study of 90 psoriasis patients and 160 controls by Torres et al. [89] using a validated tool for assessing PA, the International Physical Activity Questionnaire Short Form found that patients with severe psoriasis were 3.42 times less physically active compared to non-psoriasis patients (OR = 3,42, 95% CI: 1.47–7.9). The authors suggested that vigorous PA may protect against disease development and that the decreased engagement in exercise originates from both psychosocial and physical reasons. Similarly, the statistically significant difference in the duration of PA between psoriasis patients and controls was observed in a large cross-sectional study by Balato et al. [88]. The authors found that psoriasis patients reported a significantly shorter duration of performing sports (3–7 h/week−3.5% vs. 5.8%; >7 h/week−3.0% vs. 4.4%). In turn, a cohort study by Do et al. [90] did not confirm a significant difference in engaging in moderate-to-vigorous leisure PA between cases and controls. However, the authors found that values of equivalent metabolic tasks MET-min/week of moderate-to-vigorous PA were lower by 30% among participants with fewer or more psoriatic skin lesions than participants never diagnosed with psoriasis.

In turn, a case-control study on quality of life, stress severity, and PA based on 56 psoriasis patients (32 men, 25 women) and 36 age-matched controls without dermatoses was conducted by Nowowiejska et al. [93], using the Dermatology Life Quality Index (DLQI), the WHO Quality of Life questionnaire, and the International Questionnaire of PA. This analysis found that only 19.64% of psoriasis patients performed high-level PE, 32.14% performed moderate-level exercise, and 48.2% reported low-intensity PA (individuals not meeting the WHO criteria). These findings show that the patients with psoriasis were less physically active, took exercises of lower intensity, and felt higher stress severity and lower quality of life compared to controls.

Another experimental study by Enos [92] was based on 23 patients with psoriasis and 23 controls. The study applied questionnaires on self-efficacy for PE, pruritus, and Dermatology Life Quality Index. The authors found that patients with psoriasis preferred exercising with a 13–18% slower treadmill speed and experienced more pruritus during 20 min exercise bouts than controls. Moreover, they reported that parameters of psoriasis severity (body surface area, psoriasis area, severity index), global investigator assessment (IGA), and the Dermatology Life Quality Index showed a significant negative correlation with vigorous activity (Pearson r ranging from −0.47 to −0.62), as well as with brisk walking (Pearson r ranging from −0.47 to −0.62). The author also observed the positive correlation of the above parameters with PA levels and footsteps (Pearson r ≥ 0.60).

The recently published experimental studies listed in Table 1 confirm the previous findings that patients with psoriasis are less physically active and take up PE of a lower magnitude than healthy individuals, despite the known benefits of PA.

Evidence presents PA guidelines for the management of psoriasis. However, to date, it does not specify the exercise frequency and dose [94].

Several mechanisms may explain the beneficial effect of regular PA on psoriasis development and management. The mechanisms include energy balance, decreases in adipose tissue, prevention against overweight status/obesity, anti-inflammatory effects, and enhancement of immune function.

## 4. Limitations

The existing literature on psoriasis and PA is flawed by several limitations, which may influence the current evidence due to the complexity of this association and several influencing factors, such as age, BMI, clinical psoriasis type, and type of diet concerning dietary antioxidants. The methods used to determine PA varied between reviewed studies and could not be sufficiently reliable and valid to evaluate the effect of vigorous-intensity exercise on desired outcomes. Small sample sizes, a small amount of research focused on this topic, and consideration of a few factors as potential confounding variables in estimating the association between PE and psoriasis are another limitation, among others that are unidentified.

## 5. Conclusions

Psoriasis is an immune-mediated inflammatory disease associated with many other chronic diseases and disorders of the body’s functioning. This article demonstrates that each patient should be treated both individually and holistically. Psoriasis patients should have a healthy anti-inflammatory personalized diet and increased PA to the dose recommended by WHO. Such an approach requires the cooperation of specialists, among whom qualified nutritionists would play an important role. Based on the latest scientific reports, patients with psoriasis should have an appropriate diet tailored to their needs. The continuous development of nutrigenomics allows for implementation of new nutritional recommendations that will most likely improve the quality of life of psoriatic patients. Evidence suggests that regular-to-vigorous PA can provide physical and mental benefits to patients with psoriasis, including improved quality of life and even reduced psoriasis development. Indeed, exercise training may be an addition to diet non-pharmacologic tools, which target psoriasis. Despite the health benefits of PA, the presented reviews and the current experimental studies’ findings show that patients with psoriasis take up less vigorous PA and exercise less regularly than individuals without psoriasis. Further studies should validate the dose and timing exercise, considering the psoriasis state for disease treatment and prevention, based on observational and clinical studies with a larger sample size. The current knowledge in our review of this field may help specialists incorporate proper diet and safe exercise training for patients with psoriasis.

## Data Availability

Not applicable.
